# Systematic review of interventions for children with Fetal Alcohol Spectrum Disorders

**DOI:** 10.1186/1471-2431-9-35

**Published:** 2009-05-25

**Authors:** Elizabeth Peadon, Biarta Rhys-Jones, Carol Bower, Elizabeth J Elliott

**Affiliations:** 1 Discipline of Paediatrics and Child Health, University of Sydney, Australia; 2 Australian Paediatric Surveillance Unit, Sydney, Australia; 3 The Children's Hospital at Westmead, Sydney, Australia; 4 Telethon Institute for Child Health Research, Centre for Child Health Research, University of Western Australia, Perth, Australia

## Abstract

**Background:**

Children with Fetal Alcohol Spectrum Disorders (FASD) may have significant neurobehavioural problems persisting into adulthood. Early diagnosis may decrease the risk of adverse life outcomes. However, little is known about effective interventions for children with FASD. Our aim is to conduct a systematic review of the literature to identify and evaluate the evidence for pharmacological and non-pharmacological interventions for children with FASD.

**Methods:**

We did an electronic search of the Cochrane Library, MEDLINE, EMBASE, PsychINFO, CINAHL and ERIC for clinical studies (Randomized controlled trials (RCT), quasi RCT, controlled trials and pre- and post-intervention studies) which evaluated pharmacological, behavioural, speech therapy, occupational therapy, physiotherapy, psychosocial and educational interventions and early intervention programs. Participants were aged under 18 years with a diagnosis of a FASD. Selection of studies for inclusion and assessment of study quality was undertaken independently by two reviewers. Meta-analysis was not possible due to diversity in the interventions and outcome measures.

**Results:**

Twelve studies met the inclusion criteria. Methodological weaknesses were common, including small sample sizes; inadequate study design and short term follow up. Pharmacological interventions, evaluated in two studies (both RCT) showed some benefit from stimulant medications. Educational and learning strategies (three RCT) were evaluated in seven studies. There was some evidence to suggest that virtual reality training, cognitive control therapy, language and literacy therapy, mathematics intervention and rehearsal training for memory may be beneficial strategies. Three studies evaluating social communication and behavioural strategies (two RCT) suggested that social skills training may improve social skills and behaviour at home and Attention Process Training may improve attention.

**Conclusion:**

There is limited good quality evidence for specific interventions for managing FASD, however seven randomized controlled trials that address specific functional deficits of children with FASD are underway or recently completed.

## Background

Alcohol exposure *in utero *may impair growth, central nervous system structure and/or function and cause birth defects. Children exposed to alcohol *in utero *may have significant neurobehavioural problems persisting into adulthood [1] and/or develop one of the Fetal Alcohol Spectrum Disorders [2,3]. This spectrum includes: FAS, the most severe outcome of alcohol exposure *in utero*; Partial FAS; and Alcohol Related Neurodevelopmental Disorder (ARND), the diagnosis of which requires confirmation of maternal alcohol exposure and neurodevelopmental problems not otherwise explained. Also included are Alcohol Related Birth Defects (ARBD), the diagnosis of which require confirmation of maternal alcohol exposure and specific birth defects attributable to alcohol.[3] The term Fetal Alcohol Effects (FAE) was previously used to describe children with some, but not all of the features of FAS.[4] Adverse life outcomes for individuals with FAS or FAE include inappropriate sexual behaviours, disrupted school education, trouble with the law, confinement, and mental health, alcohol and drug problems.[5]

Early diagnosis of FAS and FAE is associated with decreased risk of adverse outcomes,[5] perhaps because it enables carers and health professionals to advocate for and deliver appropriate interventions in childhood. Interventions that have been recommended for children with FASD include pharmacological interventions (psychotropic and stimulant medications) [6,7] and educational, behavioural, social skills and communication interventions.[8,9] Carers of children with FASD report that conventional behavioural and learning approaches often fail to assist their children.[10,11]

Our aim was to systematically review the medical literature to identify and evaluate the evidence for efficacy of pharmacological and non-pharmacological interventions for children with FASD.

## Methods

### Inclusion and Exclusion Criteria, participants and outcome measures

We sought RCTs evaluating pharmacological and non-pharmacological interventions for children with FASD aged under 18 years. Non-pharmacological interventions of interest included behavioural, speech, occupational and physio therapies, early intervention programmes, and psychosocial and educational interventions. Because published reviews indicated that there were unlikely to be many RCT, we also included other study types that included a control group (quasi RCT, non-randomized controlled trials) and cohort studies with pre- and post-intervention measurements. Control interventions could include no treatment, waiting list, usual therapy or placebo.

Outcomes of interest included measures of physical and mental health, developmental status, cognitive status, quality of life, educational attainment, employment, contact with the law and substance abuse, whether measured during and immediately after the intervention and/or in adolescence and adulthood.

### Identification of studies

We searched MEDLINE (1950 – January 2009), EMBASE (1980 – January 2009), CINAHL (1982 – January 2009), PsycINFO (1865 – January 2009), Cochrane Central Register Controlled Trials (Cochrane Library Issue 1, 2009) and ERIC (1966 – January 2009), with no language restrictions, using terms for Fetal Alcohol Spectrum Disorders and therapies including: Fetal Alcohol Syndrome; Early Intervention; Drug Therapy; Allied Health Occupations; Occupational Therapy; Physical Therapy Modalities; Exercise Therapy; Behavior Therapy; and Social Support. Search terms were adapted for individual databases. Additional studies were sought by contacting individuals undertaking research on FASD and from bibliographies of identified papers, review articles and FASD conference proceedings.

### Data management and quality assessment

Two reviewers independently screened the titles and abstracts of articles identified in the search and reviewed the full text of articles that appeared to fulfill the inclusion criteria. We developed a form to standardize extraction of data regarding study design, participants, study setting, interventions and outcomes. Two independent reviewers extracted the data and assessed study quality including blinding of outcome assessment, use of standardized measures, follow-up and, for RCT, method of randomization, allocation concealment and intention-to-treat analysis (ITT). Disagreements were resolved by a third reviewer.

### Statistical Analysis

We intended to undertake meta-analyses but the data were unsuitable due to the small number of studies and the disparate interventions and outcome measures.

## Results

### Search results

**Figure 1:**
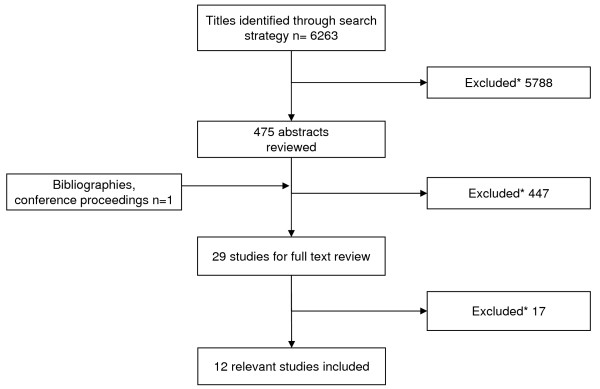
**Flow chart of the study selection process**. *Studies were excluded if they were animal studies; did not include children aged 0 to 18 years with FASD; or did not evaluate an intervention.

We identified 6263 studies using our search strategy (Figure 1). After exclusion of ineligible studies (animal studies, people with FASD aged over eighteen years and studies which did not evaluate an intervention), only twelve studies fulfilled our inclusion criteria. These included six RCT; one quasi-RCT; one controlled trial; and four pre- and post- intervention studies. Of these, two studies evaluated pharmacological interventions (total n participants = 16), seven studies evaluated educational and learning strategies (n = 167), two evaluated social skills and communication (n = 101), and one evaluated a behavioural intervention (n = 20).

### Pharmacological Interventions (Table 1)

**Table 1:** Pharmacological Interventions

**Study quality**	**Participants (n), age, inclusion criteria and setting**	**Interventions and follow-up**	**Outcome measures**
**Oesterheld et al, 1998**[12] Cross-over RCT Randomized (method unclear); allocation concealment unclear; blinding (researchers, outcome assessors); follow-up 100%; ITT analysis unclear; study power not provided.	n = 4 5 to 12 years FAS or partial FAS[4] & ADHD (DSM-IV) Native American residential school	0.6 mg/kg methylphenidate per dose to nearest 2.5 mg or lactose placebo or Vitamin C placebo Interventions were given 3 times per day for 5 days with a 2 day washout period prior to each intervention. Follow-up: Day 5 of each intervention	*Hyperactivity-Impulsivity *score on CPRS-48: significant improvement in methylphenidate group (F = 4.34, df = 2, p < 0.05) *Hyperactivity-Impulsivity *score on CTRS-39: significant improvement in methylphenidate group (F = 6.42, df2, p < 0.02) *Daydreaming-Attention *score on Conners Teacher Rating Scale-39: no significant difference (F = 1.429, df2, p = 0.289) *Adverse events: *three children experienced decreased appetite; two, mild stomach aches; and two, headaches.
**Snyder et al, 1997**[13] Cross-over RCT Randomized (method unclear); allocation concealment unclear; blinding (researchers, outcome assessors); follow-up 92%; ITT analysis unclear; study power not provided.	n = 12 6 to 16 years FAS[4] & ADHD (DSM-IV) & reported positive response to stimulant medication Selected from a child development unit database, Canada	Usual dose of medication (methylphenidate: 8 children; pemoline: 2 children; dexedrine: 1 child) or colour matched capsule (placebo) Interventions were given for 3 days with a 1 day washout prior to each intervention. Usual medication was given for 3 days between the 2 interventions. Follow-up: Day 3 of each intervention	*Attention: *No significant difference between groups on vigilance task and no significant difference on Underlining Test. *Hyperactivity: *scores on the Abbreviated Symptoms Questionnaire – Parents were significantly improved for stimulant medication (68.36, SD 17.4) compared to placebo (84.4, SD 14.0) (F = 8.66; p = 0.016) *Adverse events: *not reported

Oesterheld et al[12] randomly allocated four children to a sequence of methylphenidate and two placebos. Conner's Parent Rating Scale-48 (CPRS-48) and Conner's Teacher Rating Scale-39 (CTRS-39) were completed at the end of each day, and the Barkley Side Effects Questionnaire was completed and weight, orthostatic blood pressure and pulse rate recorded on day five of each medication. Compared to placebo, methylphenidate significantly improved hyperactivity and impulsivity but not attention. Most children receiving methylphenidate experienced adverse effects.

Snyder et al[13] randomly allocated twelve children to a sequence of usual stimulant medication and placebo. Selection bias may have occurred as the method of selection was unclear. The authors report randomization by the Pharmacy department and use of a colour matched placebo capsule which suggest possible allocation concealment and blinding of researchers and outcome assessors, but this is not clearly stated. One child was excluded from the study due to inability to complete the required tasks. Usual stimulant medication had no significant effect on performance on attention compared with placebo. Hyperactivity scores were improved significantly by stimulant medication compared to placebo.

### Educational and learning strategies (Table 2)

**Table 2:** Educational and Learning Strategies

**Study quality**	**Participants (n), age, inclusion criteria and setting**	**Interventions and follow-up**	**Outcome measures**
**Adnams et al, 2003 In: Riley et al, 2003**[14] RCT: randomization method, allocation concealment, blinding, ITT analysis unclear; follow-up 100%; study power not given.	n = 10, Mean age: 8.5 years FAS[3] Selected from previous study of 64 South African children	CCT or usual classroom, 1 hour per week, 10 school months. Follow-up: 10 months	*Behaviour: *Personal Behaviours Checklist score: improvement in CCT group compared to controls (mean pre-intervention scores 21.4 vs 14.8 and mean post-intervention scores 7.6 vs 15.4). *Neuropsychological profile: *no significant difference. *CCT battery: *qualitative improvements in function but no significant difference
**Adnams et al, 2005 In: Stromland et al, 2005**[15]** & Adnams et al, 2007**[16] RCT: randomization method, allocation concealment, ITT analysis unclear; outcome assessors blinded; follow-up 94%; study power not given.	n = 65, 9 to 10 years FAS or partial FAS[3] and "deferred diagnosis category" Exposed children selected from study of 105 South African children,	Language and literacy intervention, 1 hour per week for 38 weeks over 9 months Follow-up: 9 months	*Pre-literacy, reading and spelling: *FASD children in intervention group had significantly improved scores on Phonological Awareness and Early Literacy Test: Manipulating Syllables (t = 2.23, p = 0.034), Letter Sounds (t = 3.7, p = 0.001), Written Letters (t = 3.14, p = 0.004), Reading (t = 3.72, p = 0.001), Reading Non-Words (t = 3.65, p = 0.001) and Spelling Non-Words (t = 3.44, p = 0.002). *General scholastic tests: *No significant difference between FASD intervention and control group.
**Coles et al, 2007**[17] RCT: randomization method, allocation concealment, ITT analysis unclear; outcome assessor blinded; follow-up 100%; study power not given.	n = 32 4 to 10 years FAS or partial FAS[28], excluded if IQ < 50. Recruited from a Fetal Alcohol Clinic, USA	Virtual reality game of fire safety or virtual reality game of street safety Follow-up: immediately 1-week post-intervention	*Post-intervention: *children exposed to the computer game had significantly greater knowledge gain of fire safety (F(1, 31) = 18.94, p < 0.000) or street safety (F(1, 31) = 16.3 p < 0.000). *One week: *children exposed to the computer game had significantly greater knowledge gain of fire safety (F(1, 31) = 15.56, p < 0.000) but not street safety (F(1, 31) = 3.13, p = 0.096).
**Kable et al, 2007**[18] RCT: randomization method, allocation concealment, ITT analysis unclear; blinding (outcome assessors); follow-up 92%; sample size calculation provided.	n = 61, 3 to 10 years FAS, partial FAS, or alcohol related dysmorphology[28] Excluded if IQ < 50 or mental health problems prevented participation. Recruited: USA Fetal Alcohol Clinic and community.	Mathematics intervention (6 weeks tutoring) or a standard psycho-educational group. Follow-up: 6 weeks.	*Mathematics: *The mathematics intervention group had a significantly higher gain in mathematical knowledge (F(3, 43) = 2.97, p < 0.04) and were significantly more likely to demonstrate a clinical gain compared to the psychoeducational group (58.6 vs 23.1%, χ(1, 55) = 7.1, p < 0.008)
**Loomes et al, 2008**[19] Controlled trial Allocation method unclear; unblinded; follow up 97%; ITT analysis unclear; study power not provided.	n = 33, 4.2 to 11.8 years ARND, Alcohol Exposed Neuro-behavioural Disorder or Static Encephalopathy (criteria not stated) From hospital/FASD clinics, schools, community, Canada	Rehearsal training following pretest Follow-up: at average 10.6 days (range 6–21)	*Post-intervention: *there was no significant difference between intervention and control groups (t = -0.49, p > 0.05) *Follow-up: *the intervention group had significantly increased digit span compared to the control group (t = -1.96, p < 0.05)
**Meyer, 1998**[20] Pre-post intervention, No blinding.	n = 4, primary school age, USA. FAE & learning disabled (criteria not stated)	Four minute videotape of building task	*Learning: *No child could imitate the building block task
**Padgett et al, 2006**[21] Pre- and post-intervention; No blinding; follow-up 100%.	n = 5, 4 to 7 years FAS, partial FAS (criteria not stated); USA Fetal Alcohol Clinic	Virtual reality game of home fire safety Follow-up:1 week	*Post-intervention: *4 children correctly sequenced cards and 3 demonstrated all steps in response to an imaginary fire. *One week: *3 children correctly sequenced the cards and 5 showed all steps in response to an imaginary fire

Adnams et al reported two pilot studies evaluating educational interventions. [14-16] In the 2003 study,[14] ten children selected from a previous cohort of 64 children with FAS were randomly allocated to Cognitive Control Therapy (CCT) in an intervention classroom at one school or a control classroom at another school. The randomization method used to allocate children to the intervention or control group was not described, which means that selection bias cannot be excluded. CCT addresses body position, movement and awareness; attention; and information processing, controlling and categorizing. At baseline, the groups were similar for age, first language, socioeconomic status, school grade and locality of school and were assessed with the Cognitive Control Battery and a neuropsychological testing battery. CCT improved behaviour of the intervention group but there was no change in the control group. As the teacher rating of behaviour at baseline was worse in the intervention group at baseline, the effect of the intervention on behaviour may have been underestimated.

The 2007 Adnams et al study[15,16] recruited 65 children to evaluate language interventions focussing on basic literacy skills. Forty children with FASD were selected from a larger study of 105 children with FASD. The children with FASD were randomly assigned to language and literacy training intervention or control group. Intervention and control groups were similar in age, socioeconomic status and first language. Twenty-five children without exposure to alcohol *in utero *were randomly selected from 193 children in an epidemiologic study, and were assigned to a control group. The randomization method was not described, and selection bias cannot be excluded. Two children in the FASD control group who did not meet diagnostic criteria were excluded from analysis. Two children from the FASD intervention group and two from the alcohol unexposed control group were lost to follow up. The language and literacy intervention focussed on phonological awareness and other pre- and early literacy skills needed for reading and spelling. It was administered for half an hour, twice a week, by a speech therapist over a 9 month period for a total of 38 hours of intervention. Children were evaluated with a range of measures prior to the intervention and immediately post-intervention. The intervention led to improvements in reading, spelling and some pre-literacy domains in the FASD intervention group compared to the FASD control group. Both FASD groups continued to score lower than the alcohol unexposed control group.

Coles et al[17] randomly allocated sixteen children to exposure to a fire safety virtual reality game or a street safety virtual reality game. The randomization method was not described and selection bias cannot be excluded. Post intervention, children were tested verbally on the safety steps and were asked to act out the safety steps. The training increased the children's knowledge of fire safety and street safety.

Kable et al[18] randomly assigned 61 children to a mathematics intervention (adapted to address the neurodevelopmental difficulties seen in children with FASD) or a standard psychoeducational intervention (control). The randomization method was not described and selection bias cannot be excluded. Children were evaluated with a range of measures before the intervention and within four weeks of completing the programme. Groups were similar at baseline apart from birth weight. Two children from the intervention group and three children from the control group were lost to follow-up. The intervention group had a significantly greater improvement in mathematics knowledge.

Loomes et al[19] evaluated rehearsal training for improving memory for numbers in 33 children with FASD who were assigned to a rehearsal training group or a control group. The method of assignment to the intervention or control group was not described and selection bias cannot be excluded. The groups were similar in age at baseline. One child in the control group was lost to follow up. Both groups completed a digit span memory task at baseline, immediately post-intervention and at an average of 10.6 days following the intervention. The intervention group was given instructions on the use of rehearsal to remember information following baseline assessment and were reminded of this strategy at follow up. The intervention group had an increased score on the digit span memory task at the follow up session.

Meyer[20] evaluated modelling of perceptual tasks as a teaching strategy in four boys. Each boy was shown a video of a boy of similar age building a balanced and symmetrical structure with wooden blocks and was then given ten minutes to build the same structure with the same blocks. None of the boys was able to imitate the building project.

Padgett et al [21] evaluated the effectiveness a virtual reality game to teach home fire safety in five children. The game was tailored specifically to accommodate the usual verbal strengths and visual-spatial and fine motor weaknesses of children with FAS. Post-intervention, children were asked to sequence a set of three picture cards outlining the fire safety steps and respond to an imaginary fire in the building. The training increased the children's knowledge of home fire safety.

### Social skills and social communication interventions (Table 3)

**Table 3:** Social Communication and Behavioural Strategies

**Study quality**	**Participants (n), age, inclusion criteria and setting**	**Interventions and follow-up**	**Outcome measures**
**O'Connor et al, 2006**[22] Quasi – RCT Alternate allocation; blinding unclear; follow-up 93%; ITT analysis unclear; sample size calculation provided.	n = 100, 6 to 12 years FAS, Partial FAS or ARND[23] & social skills deficit & verbal IQ ≥ 70 Children with major sensory or motor deficits or a past diagnosis of mental retardation or pervasive developmental disorder were excluded Recruited from community, USA	CFT or delayed treatment Twelve 90-minute sessions over twelve weeks Parents attended concurrent information sessions on FASD and social skills Follow-up: 3 months	*Test of Social Skills Knowledge: *The CFT group showed significant improvement in knowledge compared to the control group (F(1, 90) = 56.52, p = 0.0001). At three months, social skills knowledge was maintained (t(48) = 1.07, p < 0.29). *Social Skills Rating System Parent : *social skills (F(1, 93) = 5.03, p < 0.03) and problem behaviours (F(1, 93) = 4.05, p < 0.05) significantly improved in the CFT group compared to the control group. At three months, parent reported social skills continued to improve (t(48) = 3.35, p < 0.002) and the decrease in problem behaviours was maintained (t(48) = 1.48, p < 0.15). *Social Skills Rating System Teacher : *no significant difference in social skills or problem behaviours between the groups immediately post-intervention or at three months.
**Timler, 2005**[25] Pre- and post-intervention Blinding unclear	n = 1, 9 years FASD[23] Recruited from a Fetal Alcohol Clinic, USA	Social communication intervention Two one-hour individual sessions per week, then four two-hour group sessions Follow-up: 6 weeks	More strategies on how to behave in a variety of social situations. Increased number of mental state verbs used.
**Vernescu, 2007**[26] RCT Randomized (method unclear); allocation concealment unclear; blinding unclear; follow up 100%; ITT analysis unclear; study power not provided.	n = 20, 6 to 12 years FASD Inuit children, Canada	Attention Process Training or contact sessions Follow-up: 3 weeks	*Measures of sustained attention: *children in the intervention group showed significant improvement. *Measures of non-verbal reasoning ability: *children in the intervention group showed significant improvement. *Measures of executive function*: no significant difference.

O'Connor et al[22] recruited 100 children to evaluate parent assisted child friendship training (CFT). A diagnosis of FASD was assigned by a paediatrician, blinded to group assignment.[23] FAS was diagnosed in 11% of the children, partial FAS in 43% and ARND in 46%. Selection bias is a potential issue as the children were allocated alternately to the intervention or delayed treatment group. The groups were similar at baseline. Four children from the intervention group and three from the delayed treatment group did not complete all the assessments. The children were assessed immediately post-intervention and at 3 months follow-up. CFT significantly improved knowledge of appropriate social behaviour. Parents reported improved social skills and fewer problem behaviours, however, teachers did not report any significant improvement. The knowledge of appropriate social behaviour, parent reported gains in social skills, and reductions in problem behaviours were maintained at three months follow-up. Children receiving neuroleptic medications alone showed significantly greater improvement on parent report of Self-control and teacher-reported Problem Behaviours.[24] Children on both stimulant and neuroleptic medication had a significantly worse outcome on teacher reported Problem Behaviours but not on parent report.

Timler[25] undertook a pre and post assessment of a social communication in one child. The intervention improved the child's social communication skills.

### Behavioural interventions (Table 3)

Vernescu[26] randomly allocated 20 children to receive Attention Process Training or control contact sessions with games and academic support. The randomization process was not described and selection bias cannot be excluded. The groups were similar at baseline. Children were assessed using measures of attention, nonverbal reasoning ability measures and behavioural measures of attention and executive function at baseline and at the end of the intervention. Teachers who completed the behavioural measures of attention and executive function were blinded to group assignment. The blinding status of the researchers and assessors of other outcome measures were not described. Children in the intervention group showed significant improvement on measures of sustained attention and non-verbal reasoning ability but there was no improvement on measures of executive function.

## Discussion and Conclusion

In this systematic review, we found limited evidence for specific interventions for children with FASD. There is evidence from RCT that a language and literacy intervention improves reading spelling and pre-literacy skills (n = 65); a mathematics intervention increases mathematics knowledge (n = 61); Attention Process Training may improve attention and non-verbal reasoning (n = 20); stimulant medication may decrease hyperactivity and impulsivity but not does improve attention (n = 16); Virtual Reality Training may facilitate learning (n = 16); and Cognitive Control Therapy in the classroom may improve behaviour (n = 10). There is evidence from a quasi-RCT of effectiveness of social skills training in improving social skills and behaviour at home but not at school (n = 100).

A strength of this systematic review is that it provides a comprehensive overview of the evidence for specific interventions for children with FASD. We searched six databases and the search was not limited by language. However, the databases included have a bias towards English language publications. A potential limitation is that we did not hand search journals. During the latter stages of our review process, a systematic review on a similar topic was published, however it differs from ours, having restricted studies to RCTs thus only including three studies.[27] By including a broader range of study types, we have been able to provide a useful summary for clinicians of the current evidence for a variety of interventions. We have also clearly identified the urgent need for more high quality intervention research. This is a rapidly evolving area with six studies published between 2006 and 2008.

The greatest limitation of our review lies in the quality of the studies available for inclusion. Significant methodological problems limit the extent to which conclusions can be drawn. Study design is often inadequate. Pre- and post-assessments and retrospective reviews are frequently used rather than RCT and in the RCT we identified, the method of randomization, allocation concealment, and blinding are rarely described. Very small sample sizes are common. Studies by O'Connor et al,[22] Adnams et al[16] and Kable et al[18] are exceptions, with sample sizes of 100, 65 and 61 respectively. The remainder of the studies have samples sizes between one and thirty-two. Small sample size may reflect challenges in recruitment and the expense of conducting intervention studies. However, a small sample size may render studies insufficiently powered to detect a true effect of treatment. In addition, the diagnostic criteria used for FASD vary between studies and sometimes are not stated. Several diagnostic criteria are described in the literature and, although similar, these have important differences.[3,4,23,28] Use of different criteria makes it difficult to compare studies outcomes because study populations may differ. It also limits the applicability of study findings to patients in other clinical settings. Another problem in the studies we identified is the short term follow-up. Disabilities associated with FASD persist into adulthood[1] so children with FASD need interventions with long term efficacy. A strength of most of the studies is the use of standardised outcome measures.

Interventions for FASD should target the specific clinical and neuropsychological deficits seen most commonly in these conditions. The neurobehavioural profile of children with FASD may include low IQ[29], however, IQ scores in individuals with FAS range from 20 to 120, and only 25% have IQ scores less than 70.[30] Children exposed to alcohol *in utero *but without the physical features of FAS may also have cognitive impairment, although this is usually less severe than in children with FAS.[31] Other common difficulties are activity and attention;[29] learning and memory;[29] language development;[29] motor abilities including balance;[29,30] visuo-spatial abilities;[29] non-verbal problem solving;[29] planning ability;[29] reaction time;[30] executive function;[32] adaptive and social skills;[5,33,34] and academic function, particularly in mathematics.[5] Many of these deficits are more severe than can be explained by IQ alone and may occur in children with IQ scores in the normal range who were exposed to alcohol *in utero*.[31]

The pattern of hyperactivity/inattention in children with a FASD diagnosis may differ from that seen in children with familial ADHD, as may their response to stimulant medications.[6] The lack of response of inattention symptoms to medication identified in this review may relate to the underlying aetiology.

One of the barriers to health professionals making a diagnosis of a FASD is their perception of a lack of effective interventions.[35,36] Nevertheless, early diagnosis of a FASD reduces the risk of developing secondary disabilities.[5] The reason for this is not clear and it may simply reflect early referral for general educational and medical support. Some studies included in our review address specific deficits of children with a FASD, including attention and social skills. Although there is currently a lack of good studies, there are seven intervention studies in progress or recently completed (Table 4)[15,37-39], A number of these are funded by the CDC[38] and appear to be high quality RCTs with adequate sample size. The forthcoming studies evaluate targeted interventions addressing specific strengths and needs of children with a FASD such as attention, behaviour and social communication and will significantly enhance the evidence base available to inform management of FASD.

**Table 4:** Studies in Progress

**Principal Investigator**	**Title**	**Study Type**	**Sample Size**
Thomas Lock[37]	Assess the Effectiveness of Atomoxetine in Children with Fetal Alcohol Syndrome and ADD/ADHD (2006 ongoing)	RCT	60
Thomas Lock[37]	Open-Label Study of the long Tern Tolerability and Safety of Atomoxetine in Children with FASD and ADD/ADHD (2006 ongoing)	Open label, uncontrolled	60
Ira Chasnoff[37,38]	Neurocognitive Habilitation for Children with Fetal Alcohol Syndrome/Alcohol Related Neurodevelopmental Disorder (2002 ongoing)	RCT	100
John Mulvihill[37,38]	Fetal Alcohol Syndrome/ARND Research Consortium: Parent Child Interaction Therapy (2001 to 2005)	RCT	100
Colleen Adnams[15]	Language and Literacy therapy, Cognitive Control Therapy and Parent Group Intervention	RCT	100
Susan Astley [37-39]	Intervening with Children/Adolescents with FAS/ARND: Positive Behaviour Support (2001 to 2005)	RCT	52
Susan Astley[38,39]	School-based social communication intervention provided directly to children with FAS/ARND	RCT	NA

NA, not available

## Competing interests

The authors declare that they have no competing interests.

## Authors' contributions

EP designed the study, conducted the search, reviewed the studies and drafted the manuscript. BRJ conducted the search, reviewed the studies and edited the manuscript. CB and EJE conceived the study, participated in the design of the study and co-wrote and edited the manuscript. All authors read and approved the final version of the manuscript.

## Pre-publication history

The pre-publication history for this paper can be accessed here:


